# Sustainable synchronized spectrofluorimetric determination of remdesivir and baricitinib: a first-derivative synchronous approach with multi-color and GLANCE assessment

**DOI:** 10.1038/s41598-026-52054-0

**Published:** 2026-06-04

**Authors:** Amal A. El-Masry, Omar A. El-Khouly, Heba Elmansi, Nahed El-Enany

**Affiliations:** 1 Department of Medicinal Chemistry, Faculty of Pharmacy, Mansoura University, Mansoura, 35516 Egypt; 2 Department of Pharmaceutical Chemistry, Faculty of Pharmacy, New Mansoura University, New Mansoura, 7723730 Egypt; 3 Department of Pharmaceutical Analytical Chemistry, Faculty of Pharmacy, Mansoura University, Mansoura, 35516 Egypt

**Keywords:** Remdesivir, Baricitinib, Synchronous spectrofluorimetry, Derivative spectroscopy, Plasma, Green analytical chemistry, Biological techniques, Chemistry

## Abstract

This study presents a novel, rapid, and sensitive spectrofluorometric method for the simultaneous quantification of remdesivir and baricitinib, for the treatment of hospitalized patients with Covid-19 pneumonia. The method utilizes a first-order derivative synchronous spectrofluorimetry technique, with measurements taken at 388 nm for remdesivir and 383 nm for baricitinib, using a wavelength interval (Δλ) of 140 nm. A linear correlation (r = 0.9999) was established between derivative signal amplitudes and concentration across the ranges of 25.0–2000.0 ng /mL for both analytes. The method was validated per ICH guidelines including accuracy and precision. The developed method also successfully quantified the target drugs individually, in laboratory prepared mixtures and laboratory-prepared dosage form, without interference from each other or from excipients. The method enabled the simultaneous analysis of these drugs in spiked human plasma at concentrations relevant to higher therapeutic levels and combination therapy studies, yielding results with acceptable precision (standard deviation ≤ 1.48). The method’s environmental sustainability and practical applicability were evaluated to confirm its overall utility for the analysis of these pharmaceuticals.

**Supplementary Information:**

The online version contains supplementary material available at 10.1038/s41598-026-52054-0.

## Introduction

The COVID-19 pandemic, instigated by the single-stranded RNA virus SARS-CoV-2, has resulted in considerable worldwide upheaval owing to its elevated transmissibility and infectiousness. Consequently, scientific endeavors have focused on formulating cures and containment techniques. Among numerous antiviral treatments, remdesivir and favipiravir have had encouraging outcomes. Both medications are thought to obstruct the viral RNA-dependent RNA polymerase enzyme, hence offering therapeutic potential in combating COVID-19^1^.

**Fig. 1:**
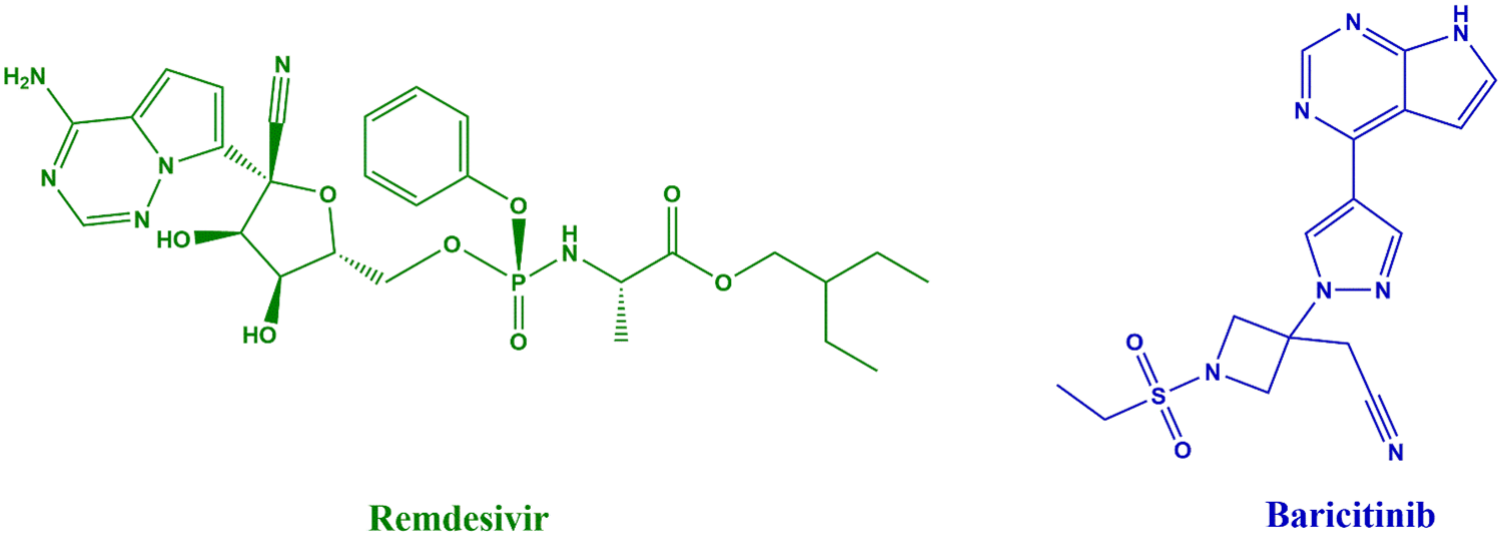
2D chemical structures of Remdesivir and Baricitinib.

Remdesivir (REM, Fig. 1), chemically characterized as 2-ethylbutyl (2S)-2-[[[(2R,3S,4R,5R)-5-(4-aminopyrrolo[2,1-f][1,2,4]triazin-7-yl)-5-cyano-3,4-dihydroxyoxolan-2-yl]methoxy-phenoxyphosphoryl]amino]propanoate^2^, interacts by inhibiting viral RNA-dependent RNA polymerase. It has exhibited *in-vitro* inhibitory efficacy against SARS-CoV-1^3^. Clinical data suggest that REM treatment may mitigate the progression to more severe respiratory illness in COVID-19 patients^4^.

Baricitinib (BAR) is 2-[1-ethylsulfonyl-3-[4-(7H-pyrrolo[2,3-d]pyrimidin-4-yl)pyrazol-1-yl]azetidin-3-yl]acetonitrile^5^. It is a disease-modifying antirheumatic medication (DMARD) sanctioned for the management of rheumatoid arthritis in adult patients with moderately to severely active conditions who have demonstrated insufficient response to standard DMARD regimens. Baricitinib is FDA-approved for the treatment of COVID-19, providing a therapeutic alternative for hospitalized patients, including paediatric instances necessitating supplementary oxygen, aggressive ventilatory therapy, or oxygenation through the extracorporeal membrane^6,7^.

Reports demonstrate that a combination therapy of BAR (an anti-inflammatory) and REM (an antiviral) was both safe and more efficacious than REM alone in the treatment of hospitalized patients with Covid-19 pneumonia. The benefits were evidenced by a one-day reduction in recovery time and enhanced clinical improvement on an ordinal rating scale^7^.

Current research presents many analytical methodologies for quantifying the antiviral medication remdesivir (REM), either alone or in combination with other drugs. The approaches encompass spectroscopy^8^, spectrofluorimetry^9,10^, HPLC^11^, and HPLC–MS^10,12^. A recent review delineates the analytical techniques established for assessing REM in biological specimens^13^ For BAR, different methods were also reported including potentiometry^14^, spectroscopy^15,16^ and HPLC methods^17^.

In recent years, the principles of Green Analytical Chemistry (GAC) have become a central pillar of modern method development, driven by the need to reduce the environmental footprint of pharmaceutical analysis^18,19^. Electrochemical and spectrophotometric techniques that emphasize the use of aqueous, non-toxic solvents, reduce reagent consumption and waste production, and do away with complicated sample preparation steps all while maintaining high analytical performance clearly demonstrate this change^20–22^. A 2026 LC article for the simultaneous determination of REM and BAR, while analytically valid, does not fully embrace these emerging sustainability principles, as it relies on organic mobile phases and generates more waste^18,23^. In contrast, the present work introduces a first-derivative synchronous spectrofluorimetric method for REM and BAR that is inherently greener. It uses water as the primary solvent, requires no organic mobile phases or complex sample preparation, and consumes minimal energy.

This work addresses key limitations of existing analytical techniques. Although spectrophotometric methods frequently lack adequate sensitivity, HPLC techniques exhibit economic and environmental disadvantages related to their significant usage of organic solvents, intricate procedures, and considerable operational expenses, potentially limiting their application in clinical environments. The described methodology enables the concurrent quantification of co-administered medications REM and BAR at nanogram levels. The spectrofluorometric method provides a more accessible option, beneficial for routine quality control and for laboratories with constrained resources. The outlined methodology facilitates the direct quantification of the REM and BAR binary mixture in diverse prepared samples without necessitating a separation step. Their simplicity and elevated sensitivity enable precise quantification of these medicines in human plasma. The method’s greenness, effectiveness, practicality, and novelty characteristics were rigorously evaluated utilizing Multi-Color Assessment (MCA) tool^24^. Furthermore, the GLANCE tool was utilized to provide an exhaustive profile of the methodological procedure^25,26^.

## Experimental

### Instrumentation, chemicals and materials

A Shimadzu RF-6000 spectrofluorometer at the Faculty of Pharmacy, New Mansoura University, New Mansoura, Egypt, equipped with a 150W xenon lamp, was utilized to obtain fluorescence spectra and measure intensity. The instrument operated at a medium voltage of 665 V, with a smoothing factor of 20, scan speed of 5 nm/s, slit width of 5 nm. Data were obtained using a delta lambda of 140 nm and smoothing factor of 20. The initial derivative synchronous amplitudes for BAR were measured at 383 nm, whereas REMs were measured at 388 nm. All pH adjustments were performed using a Jenway model 3510 pH meter (United Kingdom).

Samples of pure REM and BAR were kindly obtained by the Egyptian International Pharmaceuticals Company (EIPICO), Egypt. HPLC-grade solvents were purchased from Sigma-Aldrich (Germany). Chemicals for the formulation of various buffers were obtained from El-Nasr Pharmaceutical Chemicals Co., Egypt. Analytical-grade chemicals were utilized throughout the study. Surfactants tested (sodium dodecyl sulfate (SDS), β-cyclodextrin, and carboxymethyl cellulose (CMC)) were of analytical grade and obtained from Sigma-Aldrich (Darmstadt, Germany).

Also, Human plasma samples were obtained from the Egyptian National Blood Bank, Mansoura, Egypt, and kept frozen at −20 °C until use, then gentle thawing is performed.

### Sample and buffer preparation

Preparation of standard and working solutions:

Standard solutions of REM and BAR were prepared by transferring 10.0 mg of each drug into separate 100 mL volumetric flasks, dissolving each in 10 mL of ethanol and shaking well. The solutions were completed to the mark with the same solvent. The resulting solution concentration was 100.0 µg/mL for each drug. Serial dilution with ethanol was performed to achieve standard working solutions of 1.0 µg/mL for each drug.

A Toerell–Stenhagen universal buffer was prepared from three stock solutions:

Solution A (0.5 M H₃PO₄): 3.44 mL of 85% phosphoric acid was diluted with distilled water to a final volume of 100 mL.

Solution B (0.3 M C₆H₈O₇): 6.34 g of anhydrous citric acid was dissolved in distilled water, and the volume was completed with distilled water to 100 mL.

Solution C (1.0 M NaOH): 20 g of sodium hydroxide was dissolved in distilled water to a final volume of 500 mL.

The base buffer solution was precisely formulated by combining 100 mL of Solution A, 100 mL of Solution B, and 343 mL of Solution C. The resultant combination was then diluted to a final volume of 1000 mL with deionized water. This formulation produced a stable precursor buffer with an initial pH of about 7.0. To attain the precise target pH necessary for the analytical methods, careful modifications were made through the dropwise addition of either 0.1 M HCl or 0.1 M NaOH^27^.

### Preparation of biological samples

Plasma samples were kept frozen at −20 °C, then subjected to gentle thawing before use. 1 mL from the samples were transferred in centrifugation tubes to proceed in the development method.

### Method development and applications

#### Calibration curves

Several quantities of REM or BAR were utilized to achieve final concentrations ranging from 25.0 to 2000.0 ng/mL for both drugs. working solutions were separately transferred into 10-mL volumetric flasks, 1 mL Toerell–Stenhagen universal buffer of pH = 5 was added and diluted to volume with distilled water. Synchronous fluorescence spectra were obtained at a Δλ of 140 nm, and the resulting spectra were transformed into their first derivative utilizing Shimadzu software, Lab Solutions RF. The derivative amplitudes of REM and BAR were quantified at 388 nm and 383 nm, respectively, to develop distinct calibration curves. Linear regression equations corresponding to the data were constructed.

#### Analysis of laboratory prepared mixtures

Binary formulations of the two drugs were formulated in 10-mL volumetric flasks using distilled water, at the following concentration ratios (REM, BAR): (2:1), (4:1), and (1:1). The same method for calibration curves was followed. The % recoveries were computed to assess the accuracy and selectivity of the approach.

#### Analysis of laboratory-prepared dosage form

Ten laboratory-prepared BAR tablets (each containing 4 mg BAR, 132 mg microcrystalline cellulose (binder), 2.0 mg magnesium stearate (lubricant), 50.0 mg mannitol, and 12 mg croscarmellose sodium (disintegrant) were accurately weighed, finely powdered, and thoroughly blended for the analysis of BAR. A portion of the powdered mixture equivalent to 10.0 mg BAR and REM of the drug was precisely weighed and transferred into two separate 100.0 mL volumetric flasks. The samples were diluted with ethanol using 5o mL, then sonicated for 30 min to ensure complete extraction. Finally, the volumes were adjusted to the mark with ethanol to obtain solutions with a concentration of 100.0 μg/mL for both BAR and REM. Working solutions within the linear concentration range of the studied drugs were subsequently prepared by further dilution with ethanol. The analytical procedures under “Calibration Curves” were performed. The nominal contents of the analytes and their percentage recoveries were calculated using the respective derived regression 

equations.

#### Analysis of spiked plasma samples

For plasma sample preparation, 1.0 mL aliquots of human plasma were placed into centrifuge tubes. Each sample was then spiked with different concentrations of the drugs to achieve final concentrations within their linear ranges (REM: 50–250 ng/ mL; BAR: 50–250 ng/ mL). The solutions were properly mixed, and acetonitrile was used to precipitate plasma proteins, then adjusting the volume to 5.0 mL with acetonitrile. Following 3 min of vortex mixing to ensure complete protein precipitation, the mixtures were centrifuged at 3000 rpm for 30 min.The supernatants were passed through a 0.25 μm cellulose syringe filter, then 1.0 mL aliquot of the clear supernatant (extract) from each tube was transferred to a 10-mL volumetric flask. A blank plasma sample, derived from drug-free plasma, was processed concurrently. The analytical procedures under “Calibration Curves” were performed. Matrix-matched calibration curves were individually developed for each drug in plasma. All experiments were performed in accordance with relevant guidelines and regulations, and this work was approved by the Committee of Research Ethics in New Mansoura University, Egypt.

## Results and discussion

**Fig. 2:**
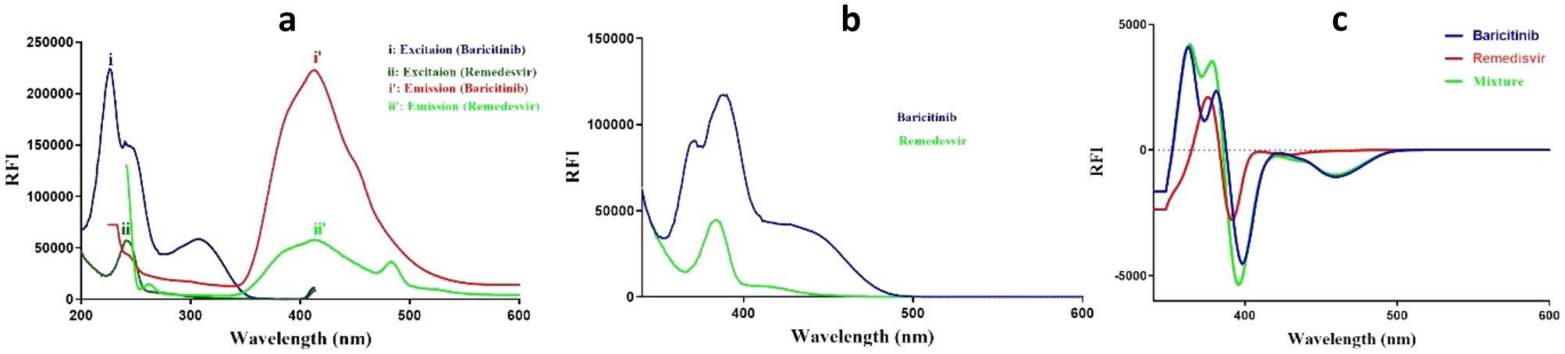
Spectral characteristics of Remdesivir and Baricitinib: (**a**) excitation and emission spectra of both drugs; (**b**) synchronous fluorescence spectra of both drugs at Δλ = 140 nm; and (**c**) first-derivative synchronous spectra of each drug individually and in their mixture.

Fluorescence is the emission of light that happens when a substance reverts to its ground state from an electrically excited state. A prevalent difficulty in the analysis of numerous medicines is the intersection of their fluorescence spectra (Fig. 2 a). This problem can frequently be efficiently addressed using synchronous spectrofluorimetry, also referred to as Stokes shift emission spectroscopy. In this technique, the excitation and emission monochromators are scanned simultaneously at the same rate, maintaining a constant wavelength difference (Δλ) between them. This method employs inexpensive solvents, and the required instrumentation is commonly found in most quality control laboratories.

The present study revealed significant spectrum overlap, even with traditional synchronous scanning, as illustrated in Fig. 2b. To address this and enhance selectivity, a sensitive method is developed based on first-derivative synchronous spectrofluorimetry. This approach successfully allowed the simultaneous estimation of remdesivir (REM) and baricitinib (BAR) without interference from each other, as illustrated in Fig. 2c.

### Optimization of different experimental parameters

Factors affecting the optimization of fluorescence intensity were carefully studied individually while the others were kept constant. These factors, including solvents, surfactants, pH, and Δλ, were systematically optimized. During the optimization study, the concentration of both drugs (BAR and REM) was maintained at 500 ng/mL.

**Fig. 3:**
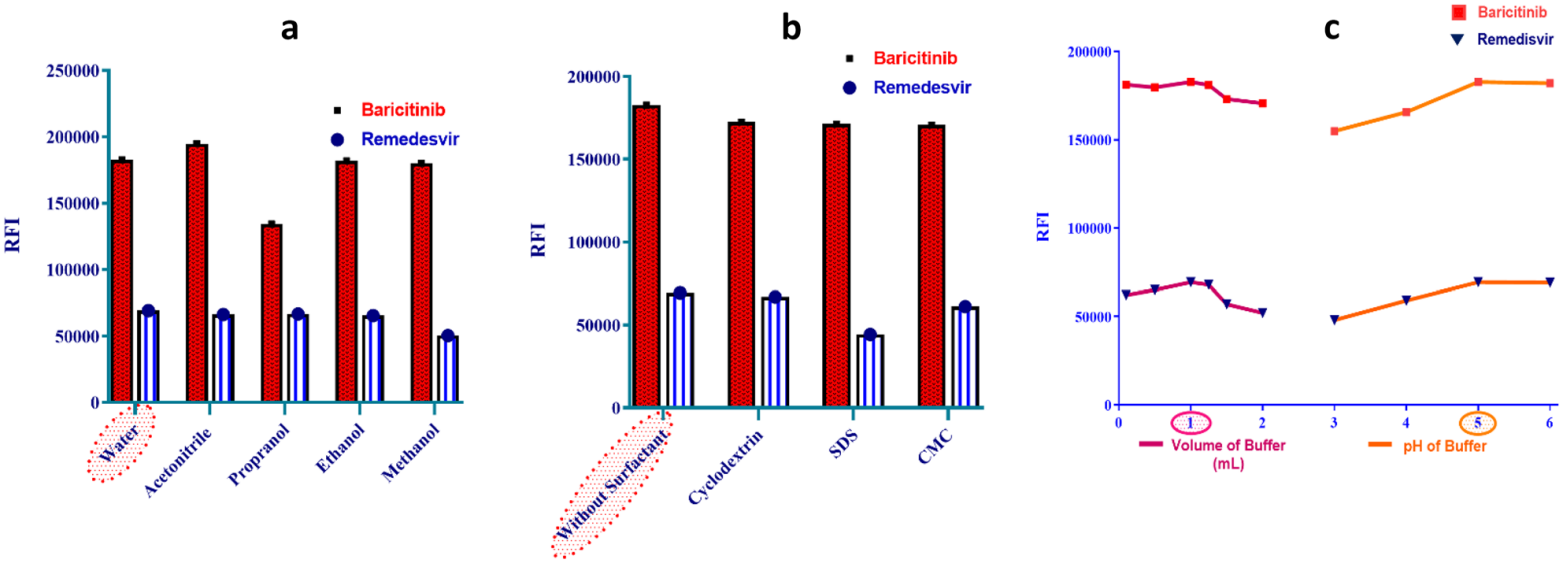
Optimization studies of factors affecting fluorescence intensity, including the effect of diluting solvents, surfactants, buffer volume, and pH. Both BAR and REM were used at 500 ng/mL.

*Solvent Selection* Various solvents (water, propanol, ethanol, methanol, acetonitrile) were evaluated for their impact on spectral resolution, blank signal, and sensitivity. Water was found to be the best solvent of choice, offering both the highest sensitivity and the greatest environmental compatibility (Fig. 3a). REM contains multiple polar functional groups including a cyano group (-CN), hydroxyl groups (-OH), a phosphate ester, and an aminopyrrolotriazine moiety. BAR features a pyrrolopyrimidine ring system, a sulfonyl group, and a nitrile group. Both analytes are capable of forming hydrogen bonds with water molecules. Water, being a highly polar protic solvent, stabilizes the excited state of these fluorophores through hydrogen bonding and dipole–dipole interactions, reducing non-radiative decay pathways and enhancing quantum yield.

*Surfactant Screening* To enhance sensitivity, various micellar media were tested above their critical micelle concentrations, including sodium dodecyl sulfate (SDS), cyclodextrin and carboxymethyl cellulose (CMC) (1 mL of 1.0% w/v each). None of these surfactants produced a significant improvement in signal (Fig. 3b). The lack of enhancement can be rationalized based on the molecular structures of REM and BAR. Both drugs possess multiple polar and ionizable groups (e.g., hydroxyl, cyano, sulfonyl, and amine moieties) that are already highly solvated in aqueous medium. The introduction of micellar hydrophobic domains may not offer additional stabilization because these analytes preferentially remain in the bulk aqueous phase rather than partitioning into the micellar core.

*pH Optimization* The Toerell–Stenhagen universal buffer, a mixture of phosphoric acid, citric acid, and boric acid adjusted with sodium hydroxide, was ultimately selected for several reasons including; wide buffering range, Compatibility with aqueous medium and reproducibility.

The effect of pH was investigated using Toerell–Stenhagen universal buffer across a range of 3 to 6. The highest performance was achieved using pH 5. Different volumes of pH 5 were also investigated, it was found that 1 mL buffer was optimum for both drugs (Fig. 3c). At pH values below 4.0, excessive protonation may lead to increased collisional quenching due to higher ionic strength and potential formation of non-fluorescent aggregates or ground-state complexes with buffer components. At pH values above 6.0, deprotonation of the ionizable groups can occur, altering the electronic distribution in the aromatic rings and often leading to bathochromic shifts but decreased quantum yield due to enhanced internal conversion and charge-transfer deactivation pathways.

*Δλ Optimization* The wavelength interval (Δλ) is a critical parameter affecting resolution, sensitivity, and spectral symmetry. Values ranging from 20 to 220 nm were meticulously analyzed. In REM, the fluorescence signal commenced at Δλ 120 nm, ascended dramatically to 140 nm, stabilized between 140 and 180 nm, and then diminished between 200 and 220 nm. For BAR, the signal was detectable from a lower Δλ of 20 nm, increased steadily until 120 nm, plateaued and remained stable at 140 nm, and then declined at higher intervals.

**Fig. 4:**
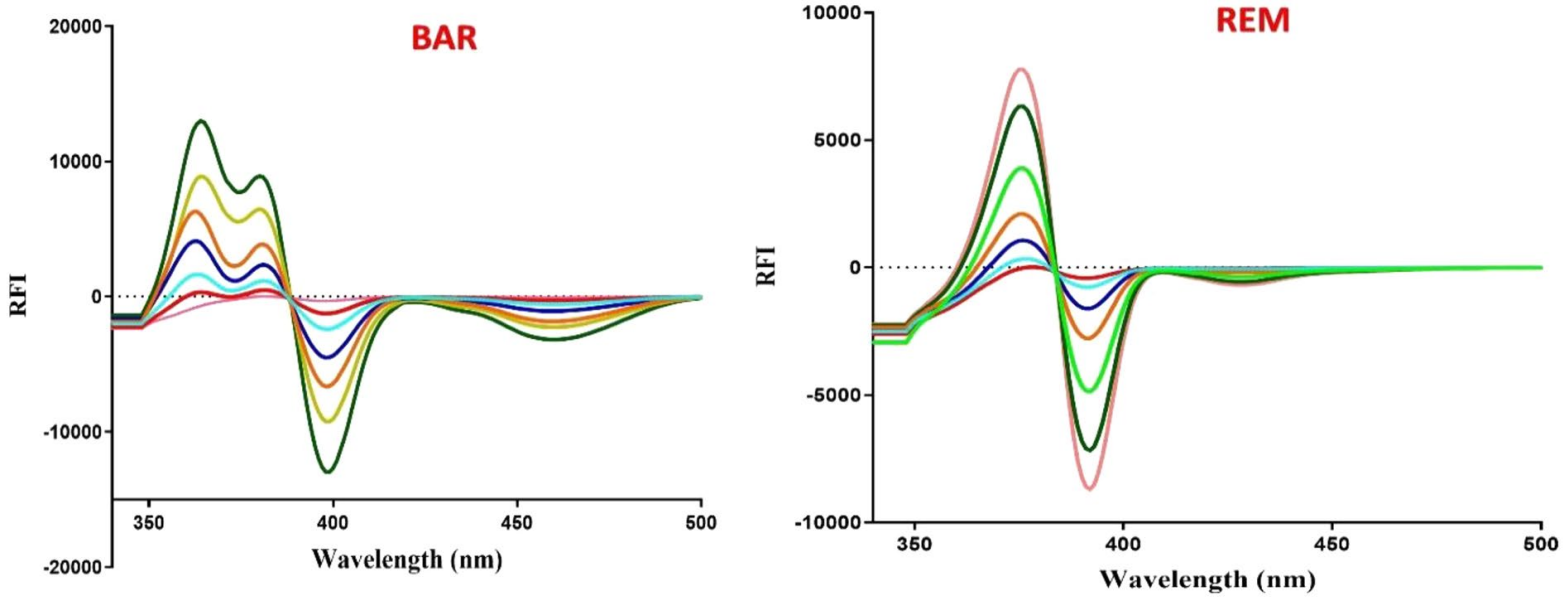
First-derivative synchronous fluorescence spectra of Remdesivir (REM) and Baricitinib (BAR) at different concentrations.

The Δλ of 140 nm constituted the optimal compromise, since it resided inside the stable, high-sensitivity plateau range for both pharmaceuticals concurrently. This configuration optimizes signal strength while maintaining symmetrical, well-defined peak forms for precise simultaneous quantification, with BAR measured at 383 nm and REM at 388 nm using first derivative synchronous amplitudes (Fig. 4).

### Validation parameters

Following optimum conditions, the method was validated according to ICH Q2(R1) guidelines^28^ as follows:

#### Linearity and range

A linear correlation was established between the first derivative amplitudes and the concentrations of each medication throughout the range of 25.0 – 2000.0, demonstrating appropriate accuracy and precision (Table 1).

**Table 1:** Performance data for the estimation of the REM and BAR by the spectrofluorometric method.

Parameter	REM	BAR
Concentration range (ng/mL)	25.0–2000.0	
Correlation coefficient	0.9999	0.9999
Slope	3.31	3.81
Intercept	344.33	59.63
LOD (ng/mL)	6.37	7.99
LOQ (ng/mL)	19.31	24.2
S_y/x_	12.95	16.44
S_a_	6.38	9.21
S_b_	0.007	0.009
% RSD	0.89	0.91
% Error = (SD / √n)	0.32	0.34

LOD: symbol for limit of determination, LOQ: symbol for limit of quantification, S_y/x_: symbol for standard deviation of residuals, S_a_: symbol for standard deviation of intercept**,** S_b_ symbol for standard deviation of slope, %RSD: symbol for a percentage of relative standard deviation.

#### Sensitivity

Limits of detection (LOD) and quantitation (LOQ) were calculated according to ICH guidelines^28^. For REM; LOD was 6.37.and LOQ was 19.31 ng/ mL. For BAR; LOD was 7.99 and LOQ was 24.2 ng/ mL. A summary of validation parameters is provided in Table 1.

#### Accuracy

Accuracy, quantified as mean percent recovery, was assessed utilizing seven concentration levels for each pharmaceutical compound. Statistical analysis of the results obtained by the proposed method and comparison methods^9,16^ revealed no significant difference between the methods regarding accuracy and precision, as shown in Table 2^29^.The two comparison methods measured each drug individually. The first method was based on measuring REM fluorescence in Britton Robinson buffer at pH 4 (λₑₓ 244 nm, λₑₘ 405 nm^8^, while the second method measured BAR fluorescence in methanol with phosphate buffer at pH 7.5 (λₑₓ 224 nm, λₑₘ 414 nm)^15^.

**Table 2:** Analytical performance comparison of the proposed method and the comparison methods for REM and BAR determination.

Drug	Proposed Method			Comparison Methods^8,15^
REM	Amount taken (ng/mL)	Amount found (ng/mL)	% Recovery^a^	% Recovery^a^
	25.0	25.31	101.25	97.45
	100.0	98.82	98.82	99.54
	250.0	249.78	99.91	101.27
	500.0	506.53	101.31	99.74
	1000.0	996.18	99.62	
	1500.0	1505.53	100.37	
	2000.0	1998.33	99.92	
${\overline{\mathrm{X}}}$ $\pm$ SD	100.17 ± 0.89			99.5 ± 1.58
t-test	0.92 (2.26)^b^			
*F*-value	3.11 (8.94)^b^			
BAR	25.0	24.53	98.12	98.83
	100.0	99.67	99.67	98.87
	250.0	247.06	98.82	101.27
	500.0	504.54	100.91	99.77
	1000.0	992.69	99.27	
	1500.0	1497.58	99.84	
	2000.0	2002.09	100.10	
${\overline{\mathrm{X}}}$ $\pm$ SD	99.53 ± 0.91			99.69 ± 1.14
*t*-test	0.25 (2.26)^b^			
*F*-value	1.6 (8.94)^b^			

^a^ Each result is the mean recovery of three separate determinations.

^b^ Figures between brackets are the tabulated *t* and F-values at (P = 0.05).

#### Intra-day and inter-day precision

The precision of the method was assessed using intra-day (repeatability) and inter-day (on three successive days) experiments at three concentration levels for each drug: 500, 1000, and 1500 ng/mL. The findings, presented as Relative Standard Deviation (RSD %), are consolidated in Table 3.

**Table 3:** Intra-day and inter-day precision data for the determination of REM, and BAR by the proposed method.

_Parameters_		REM concentration (ng/mL)			BAR concentration (ng/mL)		
		500	1000	1500	500	1000	1500
Intra-day	% Found^a^	101.31	99.61	100.37	100.91	99.27	99.84
		99.60	99.97	99.34	100.08	99.47	100.16
		100.84	100.41	98.32	99.86	100.05	99.68
	${\overline{\mathrm{X}}}$	100.58	100.0	99.34	100.28	99.60	99.89
	± SD	0.88	0.4	1.03	0.55	0.41	0.24
	% RSD	0.88	0.4	1.03	0.55	0.41	0.24
	% Error	0.51	0.23	0.6	0.32	0.24	0.14
Inter-day	% Found^a^	101.30	99.40	100.41	100.80	99.32	99.86
		99.51	98.49	99.34	100.64	99.16	100.54
		101.50	100.40	102.35	99.75	100.58	99.91
	${\overline{\mathrm{X}}}$	100.77	99.43	100.70	100.40	99.69	100.11
	± SD	1.1	0.95	1.53	0.57	0.78	0.38
	% RSD	1.1	0.96	1.52	0.57	0.78	0.38
	% Error	0.63	0.55	0.88	0.33	0.45	0.22

^a^ Each result is the mean recovery of three separate determinations.

#### Application in different matrices and selectivity evaluation

**Fig. 5:**
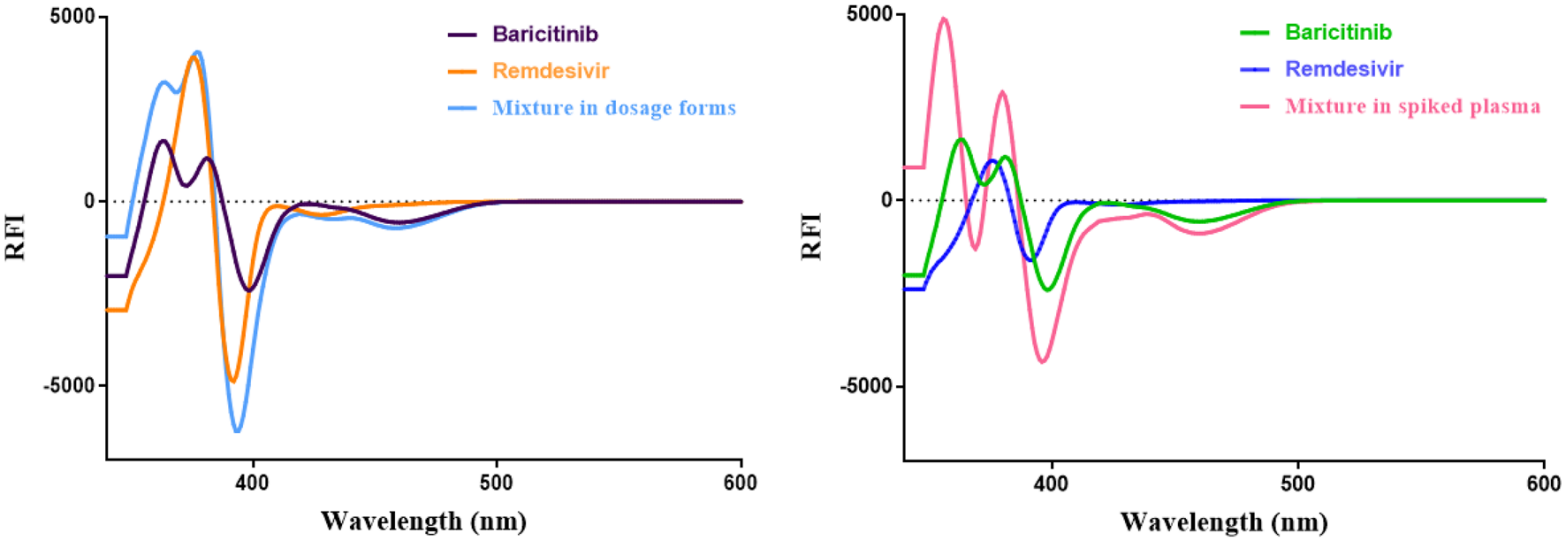
First-derivative synchronous spectra of Baricitinib and Remdesivir in their prepared dosage forms and spiked plasma.

The method’s applicability was evaluated across many matrices by assessing laboratory prepared synthetic mixtures, laboratory-prepared dosage forms, and spiked human plasma samples containing both drugs. Laboratory prepared synthetic combinations with varying concentration ratios were assessed (Table 4) and shown favourable comparisons with studies^9,16^, confirming reliable performance. The reported plasma concentrations for REM following intravenous administration are around 80.7–171 ng/mL^30^, and for BAR, in healthy subjects, a single 4 mg dose leads to a ${C}_{max}$ of approximately 24.6 ng/mL, so higher concentrations resulted after multiple doses^31^. In pediatric patients with atopic dermatitis (aged 2 to < 18 years), reported C_max_ values are higher, ranging from approximately 51.2 ng/mL to 60.4 ng/mL depending on body weight. Therefore, the selected calibration range is appropriate for this population and supports the applicability of the method within the specified biological range for BAR. Moreover, the biological concentration differs according to dose and route of administration. The suggested approach effectively measures both drugs within the specified biological ranges (Table 4). The results indicate that the approach exhibits adequate selectivity for the concurrent analysis of both drugs in these intricate matrices (Fig. 5).

**Table 4:** Determination of REM and BAR in synthetic mixture, prepared dosage form and spiked plasma by the proposed method.

Application	Concentration (ng/mL)		REM %Recovery	BAR %Recovery	Comparison Methods ^8,15^	
	REM	BAR			REM %Recovery	BAR %Recovery
Laboratory Prepared Mixture	200.0	100.0	100.84	100.03	101.54	98.4
	1000.0	250.0	100.64	98.09	100.34	99.67
	500.0	500.0	99.55	100.93	99.98	100.44
	${\overline{\mathrm{X}}}$ ^a^ ± SD	100.34 ± 0.7	99.68 ± 1.45	100.62 ± 0.82	99.5 ± 1.03	
	t-test	0.45 (2.78)^b^	0.18 (2.78)^b^			
	*F*-value	1.38 (19.0)^c^	1.99 (19.0)^c^			
Laboratory Prepared Dosage Form	200.0	100.0	102.48	101.98	100.75	98.99
	1000.0	250.0	100.15	101.43	98.31	102.32
	500.0	500.0	102.07	99.67	98.64	100.08
	${\overline{\mathrm{X}}}$ ^a^ ± SD	101.57 ± 1.25	101.03 ± 1.21	99.23 ± 1.32	100.47 ± 1.7	
	t-test	2.23 (2.78)^b^	0.47 (2.78)^b^			
	*F*-value	1.13 (19.0)^c^	1.98 (19.0)^c^			
Spiked Plasma	50.0	50.0	101.51	101.80		
	100.0	`100.0	102.17	99.96		
	250.0	250.0	99.34	99.09		
	${\overline{\mathrm{X}}}$ ^a^ ± SD	101.01 ± 1.48	100.28 ± 1.38			

^*a*^ Each result is the mean recovery of three individual analyses.

^b^ tabulated *t-*value at (*P* = *0.05*).

^c^ tabulated *F-*value at (*P* = *0.05*).

## Comparison to recent publications

A recent publication for LC determination of REM and BAR have been reported. While the recently published LC method for REM and BAR offer superior sensitivity for trace bioanalysis, our spectrofluorimetric method presents a cost-effective, greener, and operationally simpler alternative well-suited for routine pharmaceutical quality control, laboratory-prepared dosage form, and research settings where ultratrace sensitivity is not the primary requirement. The two approaches are therefore complementary rather than competitive, with each serving distinct analytical needs. A comparison between our method and the published one is summarized in Table S1.

## Comprehensive evaluation of analytical methods using the multi-color assessment tool

**Fig. 6:**
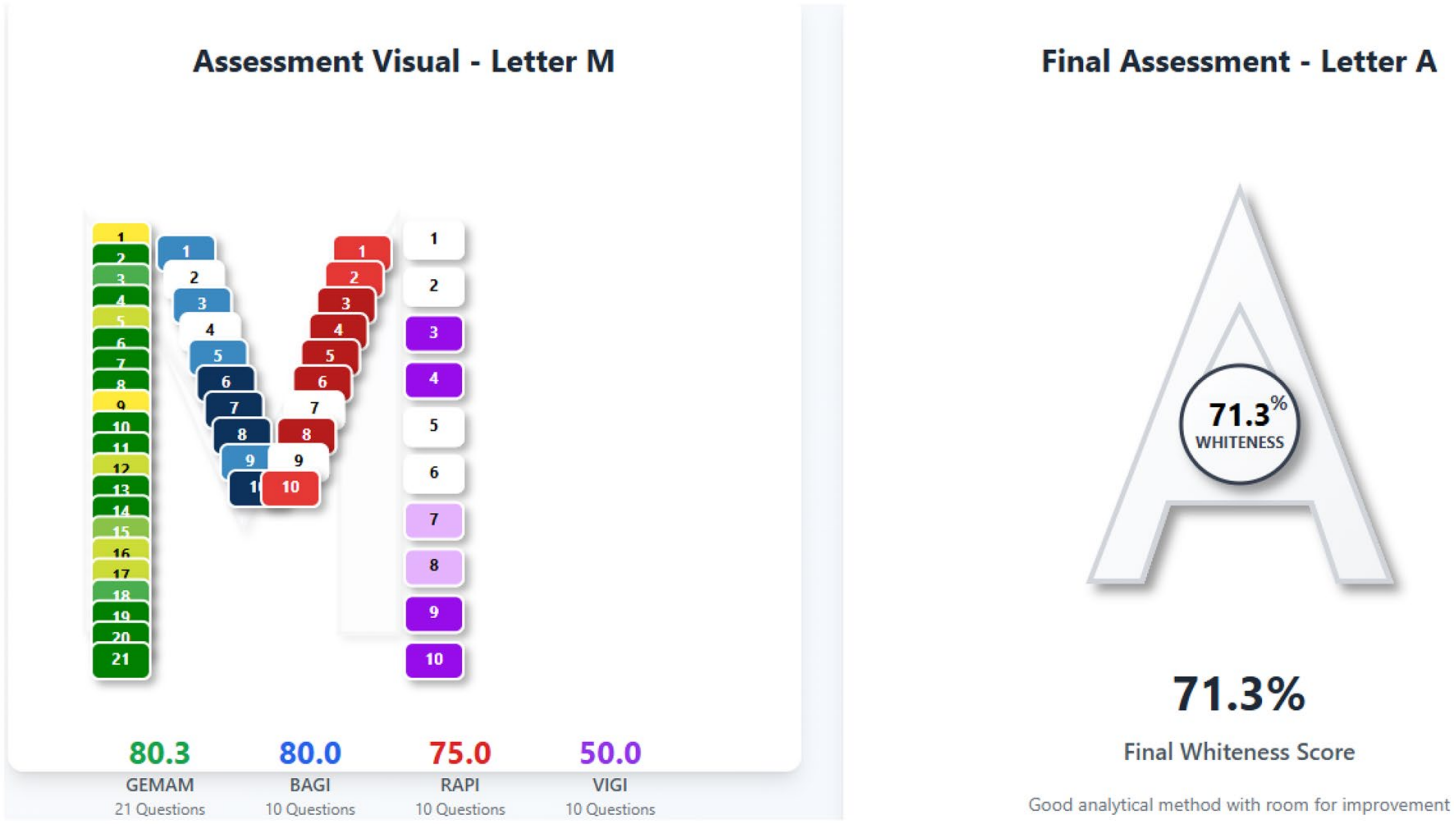
Multi-Color Assessment Visualization: (M) shows GEMAM, BAGI, RAPI, VIGI scores; while (**A**) represents overall method performance.

**Fig. 7:**
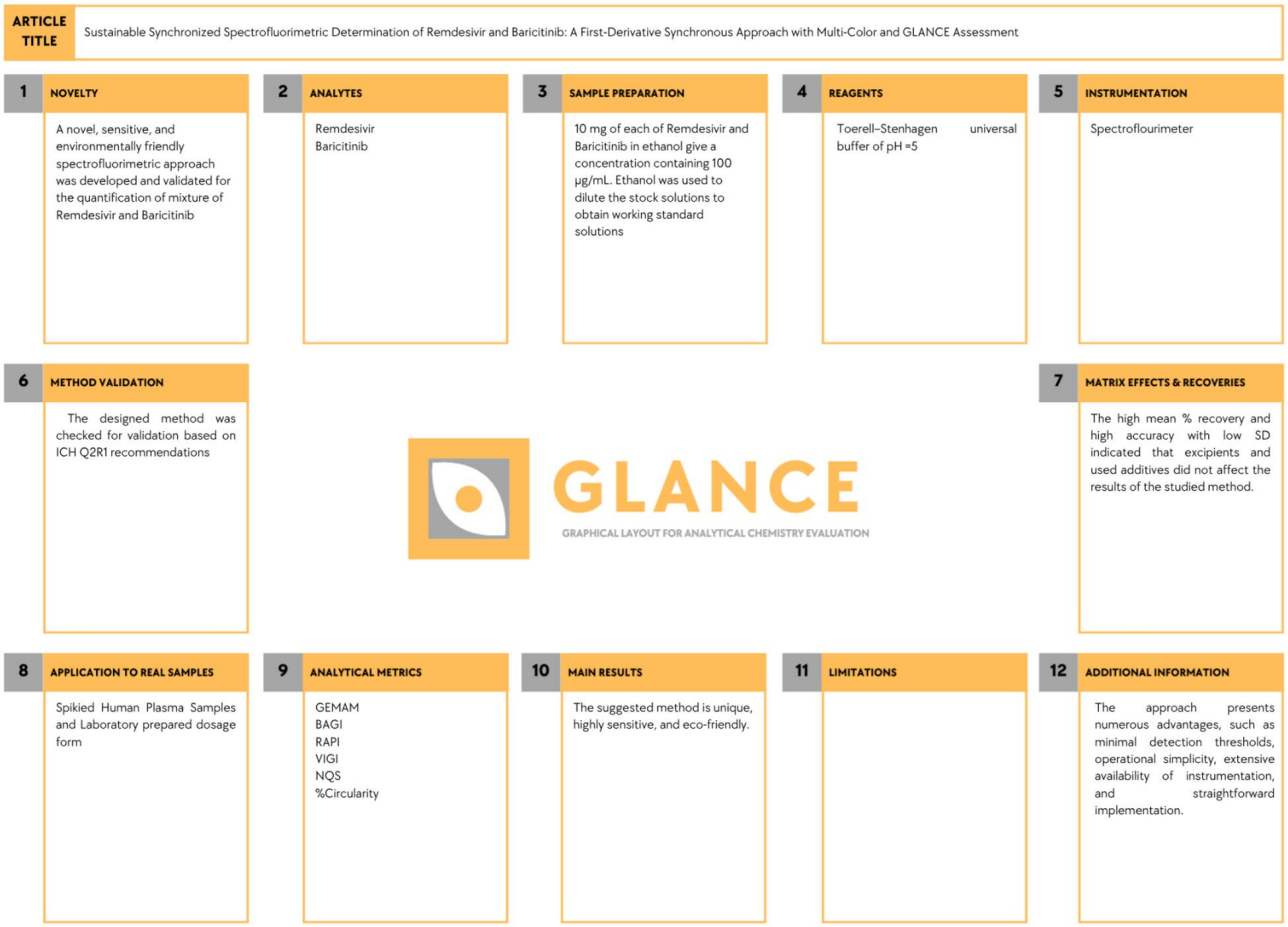
Glance aided overview of the proposed method.

The Multi-Color Assessment (MCA) tool was used as an integrated, web-based platform that combines four established evaluation frameworks into a unified assessment system^24^. The MCA tool incorporates the Green Evaluation Metric for Analytical Methods (GEMAM), Blueness Assessment Graphical Index (BAGI), Redness Analytical Performance Index (RAPI), and Violet Innovation Grade Index (VIGI) within a structured evaluation scheme consisting of fifty-one assessment questions. The tool provides graphical representations in the form of a four-segment typographic (M) and instantaneous numerical scores. These specific results are then combined to create an overall (Whiteness Score), which is represented by a stylized white (A) and represents the overall performance and sustainability of the approach. The method we employed achieved a whiteness score of 71.3%, indicating high overall sustainability, with individual scores of 80.3 for GEMAM, 80 for BAGI, 75.0 for RAPI, and 50 for VIGI (Fig. 6). An analytical method’s environmental sustainability is assessed by the 21 questions in GEMAM; as a result, a higher GEMAM value denotes a greener and more environmentally friendly method that is characterized by lower toxicity, lower energy consumption, minimized waste generation, improved safety, and better adherence to green chemistry principles. The BAGI component’s ten questions assess an analytical method’s practical applicability; therefore, a higher BAGI value reflects greater cost-effectiveness, improved time efficiency, better instrument availability, lower technical complexity, and enhanced simplicity of use, indicating strong real-world feasibility and minimal barriers to routine adoption in analytical method. The RAPI component (10 questions) evaluates how well the method performs in terms of accuracy, precision, sensitivity, selectivity, linearity, and robustness; a higher RAPI value means the method has better analytical quality and meets regulatory validation standards. The ten questions in the VIGI score assess the method’s innovation and quality, considering green chemistry aspects, automation, and modern analytical features. A score of 50 reflects a moderate but well-balanced level of innovation, indicating that the method is practical, reliable, and suitable for routine application while still incorporating essential green analytical principles. This intermediate value is not a drawback, but rather a deliberate design choice, as advanced AI- or AQbD-based approaches were not required for the scope of this study. Finally, a graphical layout tool for analytical chemistry evaluation (GLANCE) was employed to visually summarize the proposed method in a structured and clear manner (Fig. 7)^25,26^. The twelve main components of GLANCE are as follows: method novelty, targeted analytes, sample preparations, reagents used, instruments used, validation parameters (like LOD and LOQ), matrix effects and %recovery, applications, evaluation tools like greenness and blueness tools, results, limitations, and any additional information like particular protocols or precautions.

## Evaluation of sustainable development goals compliance using the need, quality, and sustainability (NQS) index

The NQS (Need, Quality, and Sustainability) index is an integrated evaluation approach designed to assess analytical methods through a multidimensional perspective that combines practical relevance, method performance, and environmental sustainability^19,32^. In contrast to conventional evaluation methods that typically emphasize a single criterion, the NQS framework adopts a multidimensional approach by integrating three complementary components: the “Need” dimension, which assesses the practical significance of the method in addressing real analytical challenges; and the “Quality” dimension, based on the White Analytical Chemistry (WAC) concept, which incorporates analytical performance, environmental friendliness, and operation, and the “Sustainability” dimension, which emphasizes environmental compatibility and alignment with the United Nations Sustainable Development Goals (SDGs). The NQS index, which combines all three components into a single normalized score, allows for direct comparison of different analytical methodologies and helps informed decision-making. This integrative approach emphasizes that an ideal analytical method should not only exhibit high sensitivity and accuracy, but also meet practical needs while minimizing environmental impact, thereby encouraging the development of more sustainable and application-oriented analytical methodologies. The percentage of need is derived by granting a 100% score to each of Koel’s four tiers. The topmost level of the pyramid corresponds to the smallest area proportion, and thus receives 25% of the overall score. if a result, the three lower-tier levels are awarded 50%, 75%, and 100%, if appropriate. As a result, a huge need at the bottom of the pyramid is calculated as 100% Need. The proposed approach required 100% of the available resources. The %Quality is calculated by taking the average of the summed percentages of redness (R), greenness (G), and blueness (B), as follows:

$$\%Quality=(\sum_{i=1}^{4}Ri+\sum_{i=1}^{4}Gi+\sum_{i=1}^{4}Bi)/3$$

The RGB 12 algorithm proposed by Nowak et al.^33^ is used to grade individual WAC elements (R1-R4, G1-G4, and B1-B4) and determine the percentage of quality (Table S1).The Quality of the proposed approach was 95.1%, while the reported method was 87.8% (HPLC-FLD) and 85.9% (LC/MS)^34^.

The alignment with the 17 SDGs is the basis for calculating % sustainability (S). It is assessed according to how well the analytical process works. The percentage of sustainability is computed by counting the agreements and dividing the result by 17. Table S2 presents the evaluation results, indicating that eight agreements were achieved.

$$\% Sustainability = 8 \times 100/17 = 47\%$$

$$\begin{gathered} So,NQS Index \left( \% \right)of the proposed method \hfill \\ = \frac{{\left( {100\% + 95.1\% + 47\% } \right)}}{3} = 80.7\% \hfill \\ \end{gathered}$$

## Circular analytical chemistry (CAC)

The Circular Analytical Chemistry (CAC) framework was used to assess the sustainability of both the proposed and reported method^35^, with comprehensive results presented in Tables S1 and S2. The proposed method displayed significant alignment, meeting nine CAC criteria and achieving an overall circularity score of 75%. In comparison, the comparison method^34^ met 6 criteria, resulting in a circularity score of 50%. It should be underlined that several CAC goals linked with broader socioeconomic and policy dimensions (Goals 10, and 11) were removed from the circularity calculation, because they became outside the direct operational domain of individual analytical methods^36^.

## Conclusion

The establishment of dependable analytical techniques for the quantification of REM and BAR as a standard procedure in COVID-19 treatment across diverse matrices is both essential and challenging. This study presents a sensitive spectrofluorimetric approach for the concurrent detection of REM and BAR. The proposed method enabled their simultaneous quantification with adequate precision and accuracy. The linear ranges for both analytes were 25–2000 ng·mL⁻^1^. Under optimized settings, water was recognized as the superior solvent, offering advantageous analytical performance and benefits of green chemistry. The findings indicate that this spectrofluorimetric method is effective for quantifying REM and BAR in synthetic mixtures, laboratory-prepared dosage form, and spiked plasma samples. The approach presents numerous advantages, such as minimal detection thresholds, operational simplicity, extensive availability of instrumentation, and straightforward implementation. The method’s environmental sustainability, efficiency, practicality, and creativity were assessed using the Multi-Colour Assessment (MCA) Tool, resulting in a whiteness score of 71.3% and robust GEMAM, BAGI, and RAPI outcomes that highlight its sustainable and effective performance.

## Supplementary Information

Supplementary Information.

## Data Availability

Raw data are available from the corresponding author upon reasonable request.
