# Supplementary material for: Sustainable synchronized spectrofluorimetric determination of remdesivir and baricitinib: a first-derivative synchronous approach with multi-color and GLANCE assessment
Source: Sci Rep. 2026 Jun 4;16:17369. doi: 10.1038/s41598-026-52054-0 (PMC13237028; doi:10.1038/s41598-026-52054-0)
Supplement: Supplementary file 1 — Supplementary Information. [file 41598_2026_52054_MOESM1_ESM.docx]

**Sustainable Synchronized Spectrofluorimetric Determination of Remdesivir and Baricitinib: A First-Derivative Synchronous Approach with Multi-Color and GLANCE Assessment**

[Amal A. El-Masr](https://royalsocietypublishing.org/doi/full/10.1098/rsos.211457)y^1*^, Omar A. El-Khouly^2^, Heba Elmansi^3^, Nahed El-Enany^2,3^

^1^Department of Medicinal Chemistry, Faculty of Pharmacy, Mansoura University, 35516 Mansoura, Egypt

^2^ Department of Pharmaceutical Chemistry, Faculty of Pharmacy, New Mansoura University, New Mansoura 7723730, Egypt

^3^ Department of Pharmaceutical Analytical Chemistry, Faculty of Pharmacy, Mansoura University, Mansoura 35516, Egypt

*Corresponding Author: [Amal](mailto:dr_heba85@hotmail.com) A. El-Masry ([**dr.Amal90@mans.edu.eg**](mailto:dr.Amal90@mans.edu.eg))

**Table S3: Comprehensive Comparison of reported method for determination of REM and BAR with the proposed method.**

| **Methods** | **Reported Method [1]** | | | | **This Method** | |
| --- | --- | --- | --- | --- | --- | --- |
|  | **HPLC-FLD** | | **LC-MS** | | **First-Derivative Synchronous** | |
| **Linearity range (ng/mL)** | BAR  0.1-50 | REM  0.5-70 | BAR  0.5-2000 | REM  0.5-1000 | BAR  25.0-2000 | REM  25.0-2000 |
| **LOD, LOQ**  **(ng/mL)** | 0.076, 0.112 | 0.167, 0.557 | 0.115, 0.382 | 0.135, 0.451 | 7.99, 24.2 | 6.37, 19.31 |
| **Applications** | Human urine and serum samples | | | | Synthetic mixtures, laboratory prepared dosage form and spiked plasma | |
| **Solvent/ Mobile phase Use** | Phosphoric acid (pH = 3) and ethanol (30:70, *v*/v) | | 0.1% Formic acid with 0.05 M ammonium formate in water and methanol (80:20, v/v) | | Ethanol and water | |
| **MA** | 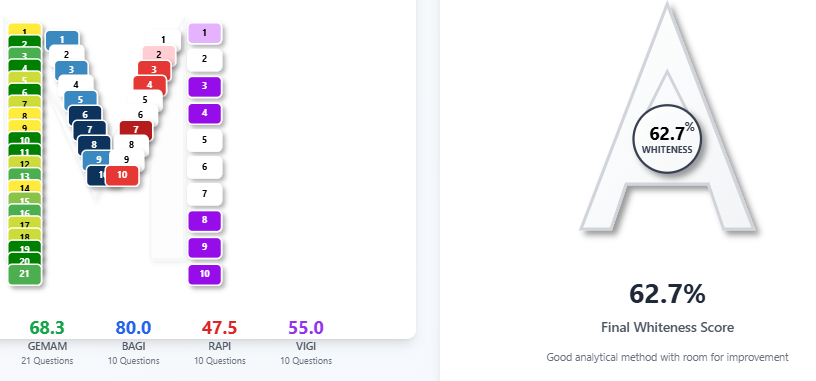 | | 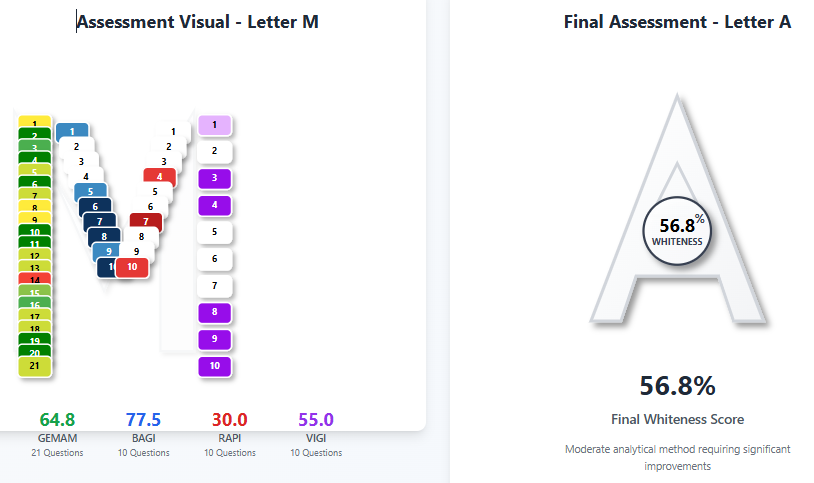 | | 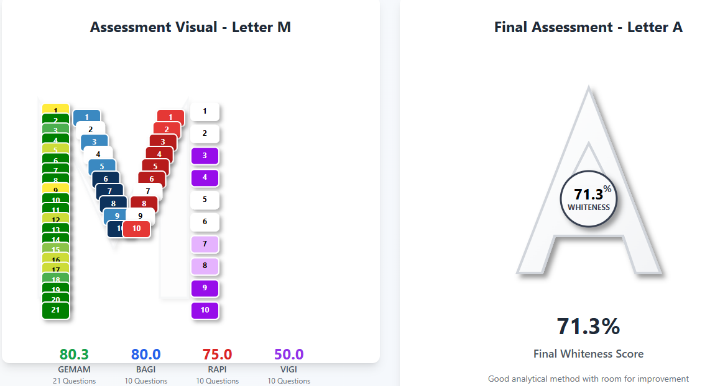 | |
| **GLANCE** | **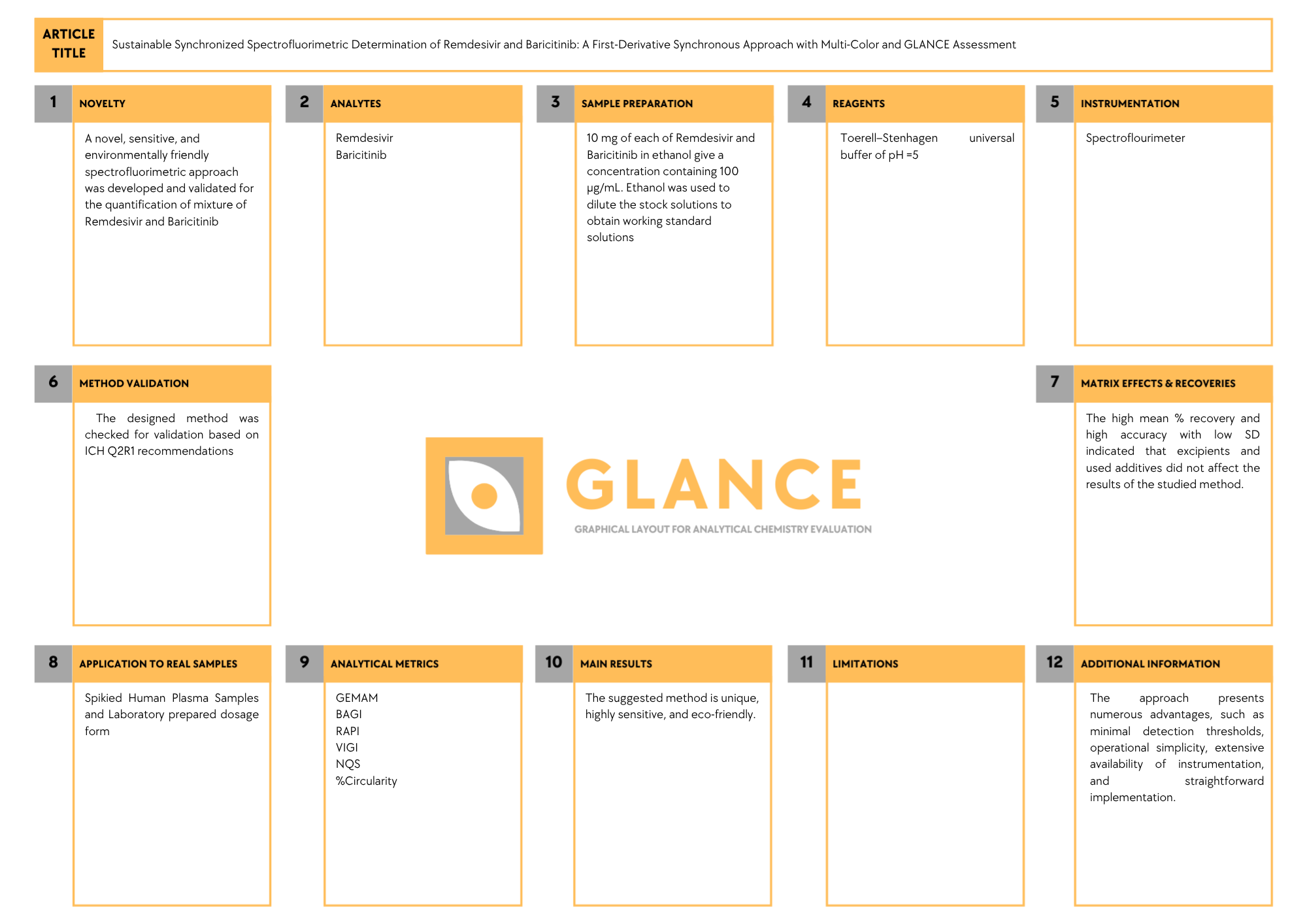**  ^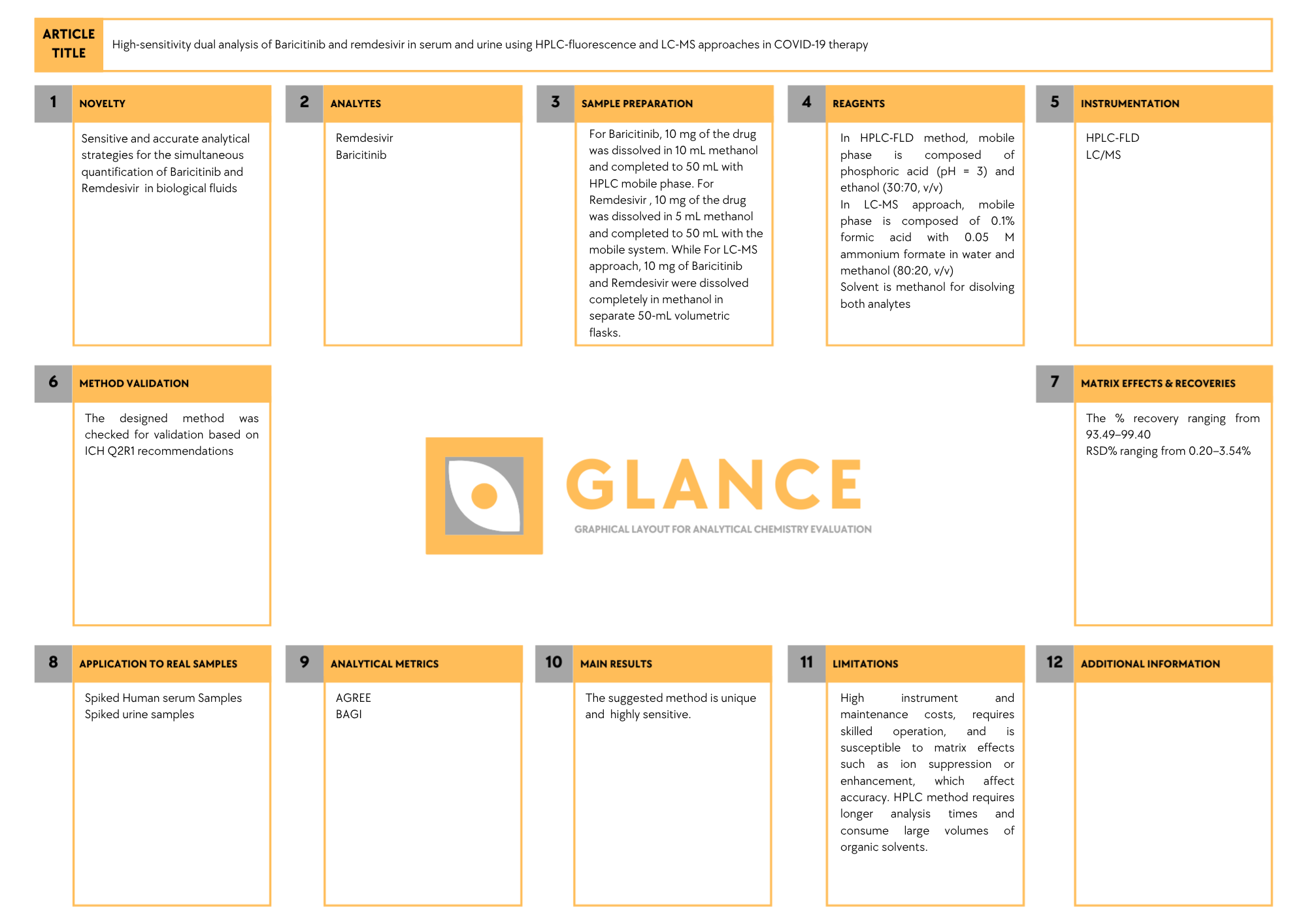^ | | | | | |
| **RGB12 algorithm** | 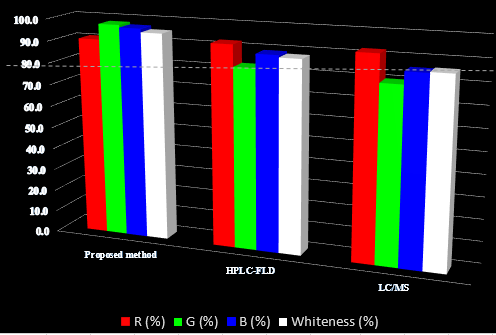  ^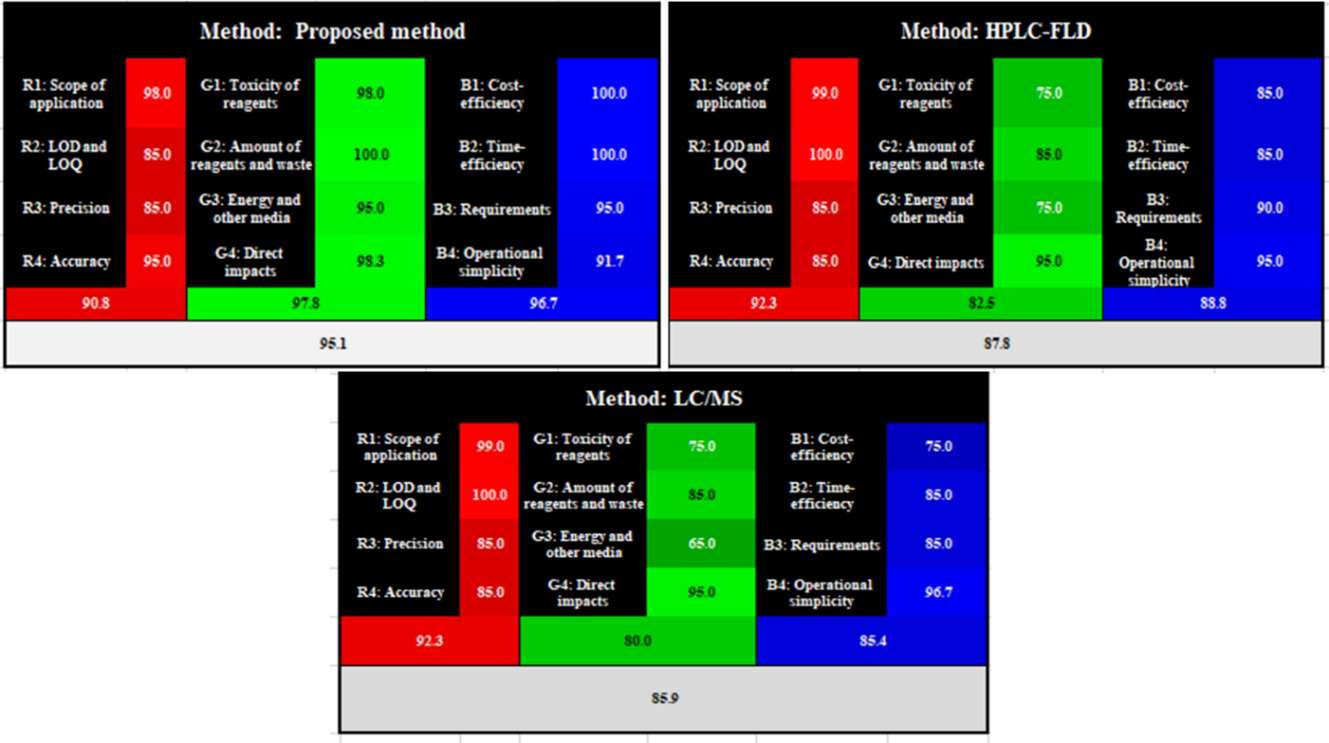^ | | | | | |
| **NQS** | $NQS Index \left( \% \right)=\left( Need\%+Quality\%+Sustanibility\% \right)/3$ | | | | | |
|  | 72.47 (HPLC-FLD)  71.77 (LC/MS) | | | | 80.7 (The proposed method) | |
| **% Circularity** | 50 | | | | 75 | |

**Table S2:** Need, Quality, and Sustainability Index (NQS) and Circular Analytical Chemistry (CAC).

|  | **Proposed Method** | **Reported method [1]** | **Remarks** | |
| --- | --- | --- | --- | --- |
| **SDG** | **Agreement** | **Agreement** |  | |
| GOAL 1: No Poverty | √ | - | The proposed method requires low instrumentation and operating costs; eliminates the need for expensive chromatographic systems, improving accessibility in resource limited laboratories. | |
| GOAL 2: Zero Hunger | - | - | No direct relevance to food or agricultural analysis. | |
| GOAL 3: Good Health and Well-being | √ | √ | Both methods enable accurate quantification of REM and BAR in biological matrices, supporting therapeutic drug monitoring and pharmacokinetic studies. | |
| GOAL 4: Quality Education | √ | √ | The procedure is simple, cost-effective, and well suited for educational use, particularly in large classrooms. it supports accessible chemistry learning by enabling safe, inclusive, and effective laboratory experiences for all students. | |
| GOAL 5: Gender Equality | - | - | No direct relevance to gender equality. | |
| GOAL 6: Clean Water and Sanitation | - | - | No direct analytical relevance. | |
| GOAL 7: Affordable and Clean Energy |  |  | No direct analytical relevance. | |
| GOAL 8: Decent Work and Economic Growth | - | - | No direct analytical relevance. | |
| GOAL 9: Industry, Innovation, and Infrastructure | √ | √ | Both approaches contribute to innovation in analytical chemistry by improving drug quantification methods, advancing laboratory capabilities, and supporting the development of more efficient, reliable methods for pharmaceutical analysis. | |
| GOAL 10: Reduced Inequality | - | - | No direct analytical relevance. | |
| GOAL 11: Sustainable Cities and Communities | √ | - | The proposed method encourages green analytical chemistry, reducing hazardous solvents, improving resource efficiency, and promoting more sustainable laboratory practices in pharmaceutical analysis. Lower solvent usage and energy demand reduce carbon footprint relative to LC-based techniques. | |
| GOAL 12: Responsible Consumption and Production | √ | - | The proposed method evolved a sustainable analytical practices through reduced chemical usage, minimized waste generation, and improved environmental efficiency in pharmaceutical drug analysis. | |
| GOAL 13: Climate Action | - | - | No direct relevance to gender equality. | |
| GOAL 14: Life Below Water | - | - | No direct relevance to gender equality. | |
| GOAL 15: Life on Land | - | - | No direct relevance to gender equality. | |
| GOAL 16: Peace and Justice Strong Institutions | √ | √ | Both methods provide accurate, validated, and reproducible results for drug determination, which strengthens regulatory compliance and ensures trustworthy data quality control laboratories and regulatory agencies | |
| GOAL 17: Partnerships to achieve the Goal | √ | √ | Both methods support interdisciplinary collaboration in pharmaceutical and clinical research. | |
| **Number of agreements** | **8** | **5** |  |  |
| **% Sustainability (Number of agreements / 17) × 100** | **47%** | **29.41%** |  |  |
| **Circular Analytical Chemistry Agreement (CAC)** | | | | |
| GOAL 1: Collect & Use Waste | **-** | **-** | Both methods generate waste, neither includes waste recovery or reuse. | |
| GOAL 2: Maximize atom circulation | √ | √ | Both are direct analytical detection methods with no chemical transformation or derivatization steps. | |
| GOAL 3: Optimize Resource Efficiency | √ | **-** | Proposed method uses minimal reagents and simpler preparation;  HPLC-FLD, LC/MS methods require mobile phases and higher consumables. | |
| GOAL 4: Strive for energy persistence | √ | **-** | Proposed method requires significantly lower energy than HPLC-FLD, LC/MS systems. | |
| GOAL 5: Enhance process efficiency | √ | √ | Both methods are rapid and validated; proposed method reduces number of analytical steps and instrument complexity. | |
| GOAL 6: No out-of-plant toxicity | √ | **-** | HPLC-FLD, LC/MS use larger volumes of organic solvents, increasing environmental burden. Proposed method is comparatively less hazardous. | |
| GOAL 7: Target optimal design | √ | √ | Both methods are optimized for sensitivity, linearity, and validation (ICH-compliant analytical design). | |
| GOAL 8: Assess sustainability | √ | √ | The proposed method is more sustainable. | |
| GOAL 9: Apply ladder of circularity | √ | √ | The proposed method has high circularity due to low reagents and energy demand. | |
| GOAL 10: Sell service, not product | **-** | **-** | - | |
| GOAL 11: Reject Lock-ins | **-** | **-** | **-** | |
| GOAL 12: Unify industry and provide coherent policy framework. | √ | √ | Both methods comply with regulatory analytical validation (ICH guidelines) supporting pharmaceutical industry standards. | |
| **Number of agreements** | **9** | **6** |  | |
| **%Circularity (No. of agreements /12)× 100** | **75** | **50** |  | |

**References:**

1- H.M. Ali, L.A. Al-Khateeb, M.M. Ghoneim, M.M. Abdelrahman, I.M. Ahmed, M. Gamal, High-sensitivity dual analysis of Baricitinib and remdesivir in serum and urine using HPLC-fluorescence and LC-MS approaches in COVID-19 therapy, Journal of Chromatography B 1272 (2026) 124927. https://doi.org/10.1016/J.JCHROMB.2026.124927.
